# Olaparib in patients with mCRPC with homologous recombination repair gene alterations: PROfound Asian subset analysis

**DOI:** 10.1093/jjco/hyac015

**Published:** 2022-02-28

**Authors:** Nobuaki Matsubara, Kazuo Nishimura, Satoru Kawakami, Jae Young Joung, Hiroji Uemura, Takayuki Goto, Tae Gyun Kwon, Mikio Sugimoto, Masashi Kato, Shian-Shiang Wang, See-Tong Pang, Chung-Hsin Chen, Tomoko Fujita, Masahiro Nii, Liji Shen, Melanie Dujka, Maha Hussain, Johann de Bono

**Affiliations:** National Cancer Center Hospital East, Chiba, Japan; Osaka International Cancer Institute, Osaka, Japan; Saitama Medical University Saitama Medical Center, Saitama, Japan; National Cancer Center, Goyang-si, South Korea; Yokohama City University Medical Center, Yokohama, Japan; Department of Urology, Kyoto University Hospital, Kyoto, Japan; Chilgok Kyungpook National University Medical Center, Daegu, South Korea; Department of Urology, Kagawa University Hospital, Kagawa, Japan; Department of Urology, Nagoya University Hospital, Nagoya, Japan; Division of Urology, Department of Surgery, Veterans General Hospital Taichung, Taichung, Taiwan; Department of Uro-oncology, Chang Gung Medical Foundation-LinKou Branch, Taoyuan City, Taiwan; Department of Urology, National Taiwan University Hospital, Taipei, Taiwan; AstraZeneca, Osaka, Japan; AstraZeneca, Osaka, Japan; Merck & Co., Inc., Kenilworth, NJ, USA; AstraZeneca, Gaithersburg, MD, USA; Division of Hematology-Oncology, Northwestern University Feinberg School of Medicine, Chicago, IL, USA; Drug Development Unit, Institute of Cancer Research and Royal Marsden Hospital, London, UK

**Keywords:** BRCA, homologous recombination repair gene alteration, mCRPC, PROfound, olaparib

## Abstract

**Background:**

The Phase III PROfound study (NCT02987543) evaluated olaparib versus abiraterone or enzalutamide (control; randomized 2:1 to olaparib or control) in men with homologous recombination repair gene alterations and metastatic castration-resistant prostate cancer whose disease progressed on prior next-generation hormonal agent.

**Methods:**

We present efficacy and safety data from an exploratory post hoc analysis of olaparib in the PROfound Asian subset. Analyses were not planned, alpha controlled or powered. Of 101 Asian patients enrolled in Japan (*n*=57), South Korea (*n*=29) and Taiwan (*n*=15), 66 and 35 patients received olaparib and control, respectively.

**Results:**

Radiographic progression-free survival (rPFS) and overall survival (OS) favored olaparib versus control in Cohort A [rPFS 7.2 vs. 4.5 months, HR 0.58, 95% CI 0.29–1.21, *P* = 0.14 (nominal); OS 23.4 vs. 17.8 months, HR 0.81, 95% CI 0.40–1.74, *P* = 0.57 (nominal)] and Cohorts A+B [rPFS 5.8 vs. 3.5 months, HR 0.69, 95% CI 0.42–1.16, *P* = 0.13 (nominal); OS 18.6 vs. 16.2 months, HR 0.96, 95% CI 0.56–1.70, *P* = 0.9 (nominal)]. Olaparib showed greatest improvement in patients harboring BRCA alterations [rPFS 9.3 vs. 3.5 months, HR 0.17, 95% CI 0.06–0.49, *P* = 0.0003 (nominal); OS 26.8 vs. 14.3 months, HR 0.62, 95% CI 0.24–1.79, *P* = 0.34 (nominal)]. Safety data were consistent with the known profile of olaparib, with no new safety signals identified.

**Conclusion:**

In PROfound, there was a statistically significant improvement in outcomes reported in the global population of patients with metastatic castration-resistant prostate cancer and alterations in homologous recombination repair genes whose disease progressed on prior next-generation hormonal agent compared with control. For the subset of Asian patients reported here, exploratory analysis suggested that there was also an improvement in outcomes versus control. The safety and tolerability of olaparib in Asian patients were similar to that of the PROfound global population.

**Clinical trial number:**

ClinicalTrials.gov NCT02987543

## Teaser

Results of the exploratory analyses presented here suggest that olaparib improved outcomes for Asian patients in the PROfound trial versus control. The safety and tolerability of olaparib in Asian patients were similar to the PROfound global population.

## Introduction

Metastatic castration-resistant prostate cancer (mCRPC) is a molecularly heterogeneous disease with a poor prognosis and limited treatment options (1–3). In addition, $\sim 1$2% of patients with mCRPC exhibit germline and somatic alterations in the homologous recombination repair (HRR) gene *BRCA1* or *BRCA2*, which is associated with particularly poor prognosis (1–3). Although prostate cancer incidence in Asia is relatively low, increasing life expectancy and an ever-growing Westernized lifestyle has resulted in a rapid rise in incidence over recent years (4). Furthermore, a higher prevalence of *BRCA1*, *BRCA2* and *ATM* alterations in the Asian population than in the Western population has been recently reported (5). However, the predominance of Western patients in many clinical trials can limit the generalizability of findings with respect to effective disease management and safety in routine clinical practice in populations of a different racial composition, particularly Asian patients. Given environmental and biological differences between Asian and Western populations, and consistent historical evidence that Asian individuals are more susceptible to the side effects of some therapeutic agents than their Western counterparts, examining the Asian population of clinical trials is of particular importance (6).

The PROfound study (NCT02987543) was a prospective, randomized, open-label, Phase III trial investigating the efficacy and safety of the poly(ADP-ribose) polymerase inhibitor (PARPi) olaparib versus physician’s choice of abiraterone or enzalutamide in men with mCRPC and alterations in at least 1 of 15 HRR genes and whose disease had progressed on prior next-generation hormonal agent (NHA; e.g. enzalutamide or abiraterone) therapy (2,7). Patient tumor tissue was tested for alterations in 15 prespecified HRR genes prospectively using an investigational clinical trial assay, based on the FoundationOne® CDx next-generation sequencing (NGS) test, in partnership with Foundation Medicine, Inc. (8). Patients were assigned to either Cohort A [*BRCA1*, *BRCA2* or *ATM* (BRCA/*ATM*)] or Cohort B (*BRIP1*, *BARD1*, *CDK12*, *CHEK1*, *CHEK2*, *FANCL*, *PALB2*, *PPP2R2A*, *RAD51B*, *RAD51C*, *RAD51D* or *RAD54L*). The study met the primary endpoint, showing that olaparib provides a statistically significant improvement in radiographic progression-free survival (rPFS) versus control (physician’s choice of enzalutamide or abiraterone) in patients with mCRPC and HRR gene alterations in Cohort A (BRCA/*ATM* alterations) (2). The study also met the prespecified secondary endpoints of rPFS in Cohorts A + B, overall survival (OS) in Cohort A, objective response rate (ORR) in Cohort A and time to pain progression in Cohort A (7). Based on the findings of the PROfound study, the US Food and Drug Administration approved olaparib for adult patients with germline or somatic HRR-gene-altered mCRPC (9) and disease progression following prior treatment with enzalutamide or abiraterone, whereas the European Medicines Agency (10), Japanese Ministry of Health, Labour and Welfare (11), Chinese National Medical Products Administration (12) and Taiwan Food and Drug Administration have approved olaparib for adult patients with *BRCA1* and *BRCA2* alterations. Regulatory review is ongoing in South Korea.

Thus far, outcome measures evaluated in the PROfound study have not been explored to determine the consistency of the clinical benefits and safety of olaparib in Asian patients. To this end, we report exploratory subset analyses to determine the efficacy and safety of olaparib versus physician’s choice of enzalutamide or abiraterone in the Asian (Japan, South Korea and Taiwan) subset of the PROfound study.

## Materials and methods

### Patients

A detailed description of the methods for the PROfound study (NCT02987543), including patient eligibility criteria, has previously been given (2). In brief, the trial included adult men with confirmed mCRPC whose disease had progressed while receiving enzalutamide or abiraterone. All patients harbored a qualifying deleterious or suspected deleterious alteration in at least 1 of 15 prespecified genes selected for their direct or indirect role in HRR. Two cohorts were enrolled: those with alterations in *BRCA1*, *BRCA2* and/or *ATM* (Cohort A), and those with alterations in ≥1 of 12 other prespecified HRR genes (Cohort B; *BRIP1*, *BARD1*, *CDK12*, *CHEK1*, *CHEK2*, *FANCL*, *PALB2*, *PPP2R2A*, *RAD51B*, *RAD51C*, *RAD51D* and *RAD54L*). Gene alterations were identified in archival or recently obtained biopsy tissue from the primary or metastatic tumor using an investigational clinical trial assay, based on the FoundationOne® CDx NGS test, developed by Foundation Medicine, Inc. Exploratory analyses for the Asian subset reported here included patients from Japan, South Korea and Taiwan.

### Trial design and interventions

Patients were randomized 2:1 to receive olaparib tablets (300 mg twice daily; *n* = 256) or control [prespecified physician’s choice of enzalutamide (160 mg once daily) or abiraterone (1000 mg once daily) plus prednisone (5 mg twice daily; prednisolone is permitted for use instead of prednisone in Japan); *n* = 131]. Randomization was stratified according to previous taxane use (yes or no) and measurable disease (yes or no). Patients who were assigned to the control group were allowed to cross over to receive olaparib treatment after independent review-confirmed imaging-based progression if they had not received any subsequent anticancer therapy following discontinuation of randomized treatment and any unresolved toxicities from prior therapy were controlled and were grade ≤ 1 at the time of initiating olaparib treatment (verified by blinded independent central review if it occurred before the primary analysis data cut-off date of 4 June 2019, or by site investigator review if it occurred thereafter). All patients provided written informed consent to participate.

### Endpoints

The primary endpoint of the PROfound study, rPFS in Cohort A for the intention-to-treat (ITT) population, has been reported previously (2). Key secondary endpoints, including rPFS in the overall population (Cohorts A + B) and OS, ORR [defined as the percentage of patients who had an imaging-based complete response or partial response by Response Evaluation Criteria in Solid Tumors (RECIST), version 1.1] and time to pain progression in Cohort A, have also been reported previously (2,7).

Exploratory analyses for the Asian subset of patients reported here include rPFS, ORR, OS and safety. Response rate was assessed among patients who could be evaluated and who had measurable disease at baseline, as assessed by independent review according to RECIST.

### Safety

Safety findings in the global PROfound population have been reported previously (2). The safety analysis set comprised all randomized patients from Cohorts A + B who had received at least one dose of assigned therapy. Safety was assessed through reporting of adverse events (AEs) according to the Common Terminology Criteria for Adverse Events, version 4.0 (2,7).

### Statistical analysis

A detailed description of the statistical methods used in the PROfound study have been described previously (2); the same methods were used in the analyses of the Asian subset.

In brief, rPFS by blinded independent central review and OS were analyzed using a stratified log-rank test, and related hazard ratios (HRs) and 95% confidence intervals (CIs) were calculated using the Cox proportional hazards model. Kaplan–Meier curves were used to estimate medians. Confirmed radiological ORR by blinded independent central review was analyzed by logistic regression.

All analyses of the Asian subset were exploratory and not powered for significance. The data cut-off date for the primary analysis of rPFS and ORR was 4 June 2019. The data cut-off date for the final analysis of OS, subsequent therapy (crossover data) and safety data was 20 March 2020.

This trial was performed in accordance with the principles of the Declaration of Helsinki, Good Clinical Practice guidelines, and the AstraZeneca and Merck policies on bioethics.

## Results

### Patients

Of the 387 patients in the global study population, 101 Asian patients were enrolled at sites in Japan (*n* = 57), South Korea (*n* = 29) and Taiwan (*n* = 15). Within the Asian subset, 66 and 35 patients were allocated to the olaparib and control arms, respectively. Fifty-nine patients were assigned to Cohort A and 42 to Cohort B. Within the Asian subset, 34 patients had a BRCA alteration, of whom 25 were randomized to olaparib and 9 to physician’s choice of abiraterone or enzalutamide. A full overview of the HRRm detected in the Asian subset patients is shown in the Supplementary Appendix (Table S1).

Demographics and baseline characteristics of the Asian subset of patients were generally balanced between treatment arms and consistent with those of patients in the global ITT population (Table 1). However, compared with the global ITT population, Asian patients in the olaparib arm had a lower median weight (68.2 vs. 78.0 kg), higher rate of metastatic disease at initial diagnosis (62.1% vs. 25.7%), higher median prostate-specific antigen (PSA) at baseline (167.0 vs. 68.2 μg/L) and higher rate of visceral metastasis (36.3% vs. 26.6%).

**Table 1:** Baseline characteristics of the Asian subset of patients and overall population enrolled in PROfound

Characteristic	Asian subset Cohorts A + B		Overall Cohorts A + B	
	Olaparib	Control	Olaparib	Control
	(*N* = 66)	(*N* = 35)	(*N* = 256)	(*N* = 131)
Median age at randomization, years (range)	72 (47–85)	69 (55–80)	69 (47–91)	69 (49–87)
Age ≥ 65 years at randomization, *n* (%)	49 (74.2)	30 (85.7)	174 (68.0)	97 (74.0)
Median weight, kg	68.2	64.6	78.0	76.6
Metastatic disease at initial diagnosis, *n* (%)	41 (62.1)	25 (71.5)	66 (25.8)	25 (19.1)
Gleason score ≥ 8,^a^ *n*/total *n* (%)	56/66 (84.8)	30/35 (85.7)	183/251 (72.9)	95/127 (74.8)
ECOG performance status 0/1, *n* (%)	64 (97.0)	35 (100.0)	243 (94.9)	126 (96.2)
Median PSA at baseline, μg/L (IQR)	167.0 (38.7–413.5)	67.0 (28.7–255.8)	68.2 (24.1–294.4)	106.5 (37.2–326.6)
Metastases at baseline, *n* (%)				
Bone only	14 (21.2)	15 (42.9)	86 (33.6)	38 (29.0)
Visceral: lung or liver	24 (36.3)	4 (11.5)	68 (26.6)	44 (33.6)
Other	13 (19.7)	3 (8.6)	88 (34.4)	41 (31.3)
Previous NHA, *n* (%)				
Enzalutamide only	31 (47.0)	14 (40.0)	105 (41.0)	54 (41.2)
Abiraterone only	15 (22.7)	12 (34.3)	100 (39.1)	54 (41.2)
Enzalutamide and abiraterone	19 (28.8)	9 (25.7)	51 (19.9)	23 (17.6)
Previous taxane use, *n* (%)				
Docetaxel only	33 (50.0)	14 (40.0)	115 (44.9)	58 (44.3)
Cabazitaxel only	2 (3.0)	0	3 (1.2)	0
Docetaxel and cabazitaxel	9 (13.6)	6 (17.1)	51 (19.9)	26 (19.8)

ECOG, Eastern Cooperative Oncology Group; IQR, interquartile range; NHA, next-generation hormonal agent; PSA, prostate-specific antigen.

^a^In general, Gleason scale ranges from 6 to 10, with higher scores indicating worse prognosis.

### Efficacy

#### PFS

**Figure 1:**
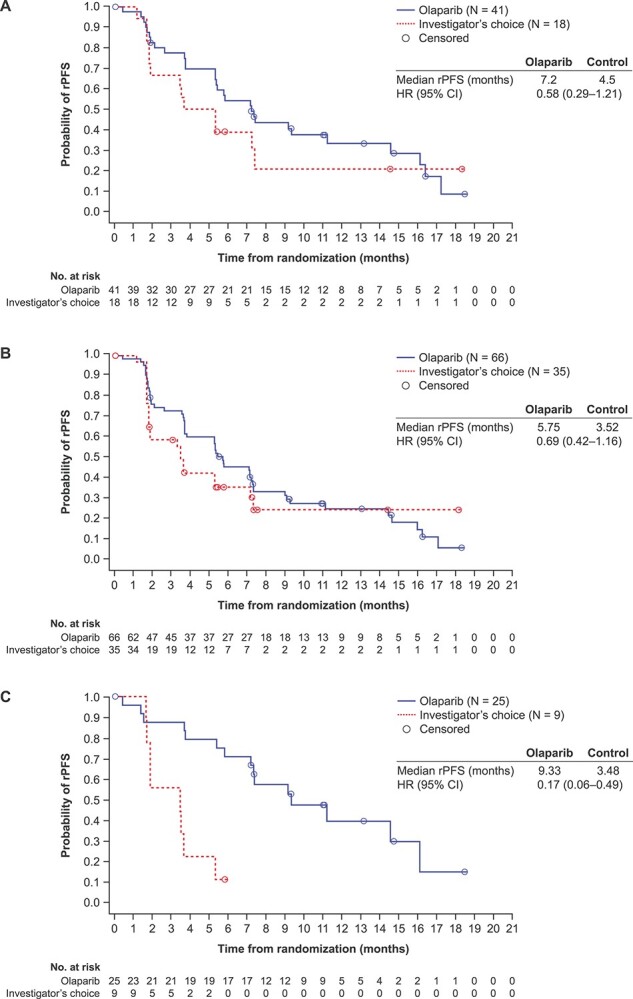
Radiographic progression-free survival in the Asian subset of patients in (A) Cohort A, (B) Cohorts A + B and (C) patients with BRCA alterations (Cohorts A + B^a^). ^a^Cohorts A + B as two patients with co-occurring alterations, including alterations in *BRCA2*, were incorrectly assigned to Cohort B. CI, confidence interval; HR, hazard ratio; rPFS, radiographic progression-free survival.

rPFS was numerically longer in the olaparib arm than in the control arm in Asian patients in Cohort A [median 7.2 vs. 4.5 months; rPFS HR 0.58, 95% CI 0.29–1.21, *P* = 0.14 (nominal); Fig. 1A] and the overall Asian subset [Cohorts A + B; median 5.75 vs. 3.52 months; rPFS HR 0.69, 95% CI 0.42–1.16, *P* = 0.13 (nominal); Fig. 1B]. In Asian patients with BRCA alterations, rPFS was numerically longer following olaparib treatment than with control [median 9.33 vs. 3.48 months; rPFS HR 0.17, 95% CI 0.06–0.49, *P* = 0.0003 (nominal); Fig. 1C].

#### ORR

Among evaluable Asian patients in Cohort A, ORR was 36.8% (7/19 patients) in the olaparib arm and 0% (0/6 patients) in the control arm. In the overall Asian subset (Cohorts A + B), ORR was 22.2% (8/36 patients) in the olaparib group and 0% (0/14 patients) in the control group, and in Asian patients with BRCA alterations, ORR was 50.0% (7/14 patients) in the olaparib group and 0% (0/4 patients) in the control group. Owing to the small numbers of patients and events, odds ratios were non-calculable.

#### OS

**Figure 2:**
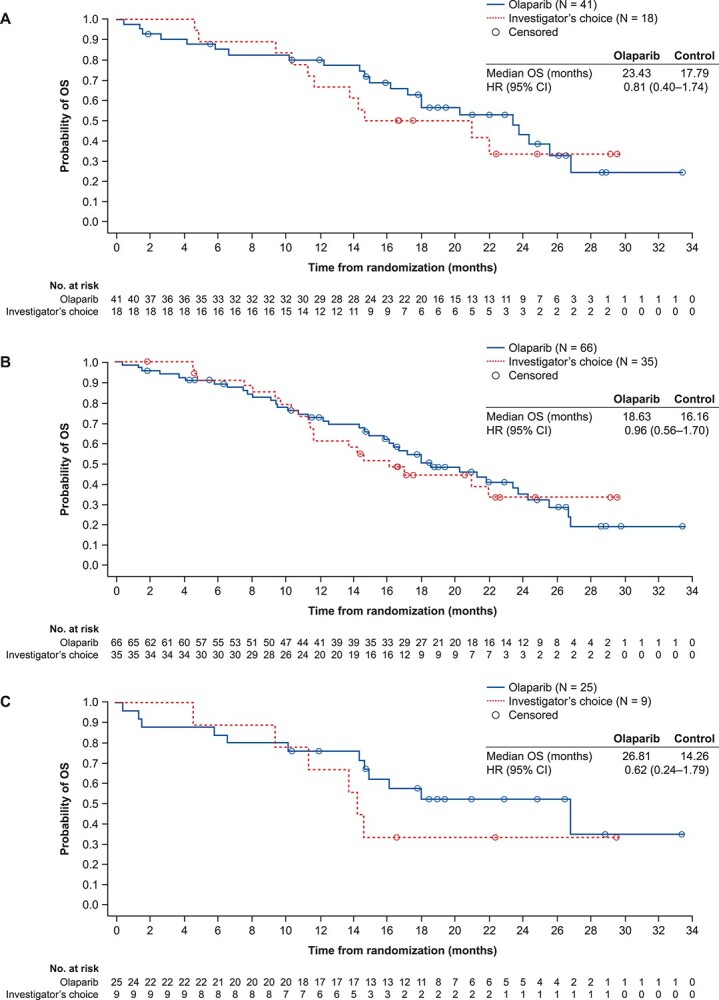
Overall survival in the Asian subset of patients from PROfound: (A) Cohort A, (B) Cohorts A + B and (C) patients with BRCA alterations (Cohorts A + B^a^). ^a^Cohorts A + B as two patients with co-occurring alterations, including alterations in *BRCA*2, were incorrectly assigned to Cohort B. CI, confidence interval; HR, hazard ratio; OS, overall survival.

Median OS was 23.4 months in the olaparib arm and 17.8 months in the control arm in Asian patients in Cohort A [OS HR 0.81, 95% CI 0.40–1.74, *P* = 0.57 (nominal); median follow-up was 19.4 and 22.4 months for the olaparib and control arms, respectively; Fig. 2A] and 18.6 and 16.2 months, respectively, in the overall Asian subset [Cohorts A + B; OS HR 0.96, 95% CI 0.56–1.70, *P* = 0.9 (nominal); median follow-up was 18.8 and 17.5 months for the olaparib and control arms, respectively; Fig. 2B]. A similar trend was observed in Asian patients with BRCA alterations [26.8 vs. 14.3 months; OS HR 0.62, 95% CI 0.24–1.79, *P* = 0.34 (nominal); median follow-up was 19.4 and 22.4 months for the olaparib and control arms, respectively; Fig. 2C].

More Asian patients in the control arm tended to cross over compared with the global ITT population. Among patients in the control arm with independent review-confirmed imaging-based progression, the number of patients who switched to olaparib in Cohort A, the overall Asian subset (Cohorts A + B) and Asian patients with BRCA alterations was 11 (61%), 24 (69%) and 6 (67%), respectively.

## Safety analysis

Median duration of treatment exposure in the subset of Asian patients was 8.7 months in the olaparib arm and 4.1 months in the control arm. In the global study population, median duration of treatment exposure was 7.6 months for the olaparib arm and 3.9 months for the control arm.

The safety and tolerability profile of olaparib in Asian patients was broadly consistent with that reported previously in the global PROfound study population (Table 2), although the number of patients with discontinuations due to an AE was smaller for Asian patients in the olaparib arm than in the global study population (12.1% vs. 19.9%, respectively).

**Table 2:** Safety summary in the Asian and overall study populations

AE, *n* (%)	Asian subset	Overall		
	Olaparib (*n* = 66)	Control (*n* = 35)	Olaparib (*n* = 256)	Control (*n* = 130)
Any AE	60 (90.9)	27 (77.1)	246 (96.1)	115 (88.5)
Any AE of CTCAE grade ≥ 3	37 (56.1)	10 (28.6)	133 (52.0)	52 (40.0)
Dose reduction due to AE	13 (19.7)	1 (2.9)	60 (23.4)	7 (5.4)
Discontinuation due to AE	8 (12.1)	2 (5.7)	51 (19.9)	11 (8.5)
Death due to AE	2 (3.0)	1 (2.9)	10 (3.9)	6 (4.6)
Reported to be related to study treatment	0	0	1 (0.4)	1 (0.8)

AE, adverse event; CTCAE, Common Terminology Criteria for Adverse Events, version 4.0.

The incidence of any AE or grade ≥ 3 AEs in Asian patients was generally consistent with that in the global study population (Table 3). In the olaparib arm, anemia and nausea were the most commonly reported AEs among Asian patients (45.5% and 33.3%, respectively) and the global study population (49.6% and 43.0%, respectively). In the olaparib arm, the AEs reported less frequently by Asian patients versus the global study population (>10% difference between subset and global study population) were constipation (7.6% vs. 19.1%) and diarrhea (7.6% vs. 21.5%). In the olaparib arm, the greatest contrast in incidence of grade ≥ 3 AEs between Asian patients and the global study population were for decreased white blood cell count (6.1% vs. 1.6%), although this was not the case for neutropenia (1.5% vs. 3.9%).

**Table 3:** Adverse events in the PROfound Asian subset and overall population (Cohorts A + B)

AE, *n* (%)	Asian subset				Overall			
	Olaparib		Control		Olaparib		Control	
	(*n* = 66)		(*n* = 35)		(*n* = 256)		(*n* = 130)	
	All grades	Grade ≥ 3	All grades	Grade ≥ 3	All grades	Grade ≥ 3	All grades	Grade ≥ 3
Any AE	60 (90.9)	37 (56.1)	27 (77.1)	10 (28.6)	246 (96.1)	133 (52.0)	115 (88.5)	52 (40.0)
Anemia	30 (45.5)	17 (25.8)	2 (5.7)	0	127 (49.6)^a^	58 (22.7)	20 (15.4)	7 (5.4)
Nausea	22 (33.3)	1 (1.5)	4 (11.4)	0	110 (43.0)	4 (1.6)	27 (20.8)	0
Decreased appetite	19 (28.8)	2 (3.0)	4 (11.4)	0	80 (31.3)	4 (1.6)	24 (18.5)	1 (0.8)
Vomiting	11 (16.7)	1 (1.5)	3 (8.6)	0	51 (19.9)	6 (2.3)	17 (13.1)	1 (0.8)
Cough	9 (13.6)	0	0	0	29 (11.3)	0	3 (2.3)	0
Malaise	4 (6.1)	1 (1.5)	4 (11.4)	0	5 (2.0)	1 (0.4)	6 (4.6)	0
Constipation	5 (7.6)	0	5 (14.3)	0	49 (19.1)	0	19 (14.6)	0
Diarrhea	5 (7.6)	1 (1.5)	2 (5.7)	0	55 (21.5)	2 (0.8)	9 (6.9)	0
Dizziness	6 (9.1)	0	2 (5.7)	0	18 (7.0)	0	5 (3.8)	0
Fatigue	4 (6.1)	1 (1.5)	4 (11.4)	0	69 (27.0)	4 (1.6)	28 (21.5)	3 (2.3)
White blood cell count decreased	8 (12.1)	4 (6.1)	0	0	12 (4.7)	4 (1.6)	0	0
Back pain	5 (7.6)	0	1 (2.9)	1 (2.9)	36 (14.1)	2 (0.8)	18 (13.8)	2 (1.5)
Nasopharyngitis	5 (7.6)	0	2 (5.7)	0	7 (2.7)	0	4 (3.1)	0
Osteonecrosis of jaw	5 (7.6)	1 (1.5)	2 (5.7)	0	5 (2.0)	1 (0.4)	3 (2.3)	1 (0.8)

AE, adverse event.

^a^Anemia (49%) and decreased hemoglobin (<1%).

## Discussion

These exploratory analyses suggest that Asian patients with mCRPC and alterations in HRR genes and whose disease had progressed on prior treatment with NHA had clinically meaningful rPFS and OS improvements when treated with olaparib compared with abiraterone or enzalutamide.

The primary endpoint of median rPFS was longer, and HR directionally favored olaparib, in patients with alterations in Cohort A (*BRCA1*, *BRCA2* or *ATM*). Similarly, median OS favored olaparib in both the overall Asian subset and the subset of Asian patients with BRCA alterations. It should be noted that there are limitations to these analyses as PROfound was not powered to detect a treatment effect across any subset, and some subsets in these analyses were small and were not adjusted for factors such as differences in baseline characteristics or rate of crossover. However, although the nominal *P* values do not have statistical power, overall, the Asian subset analysis demonstrated similar trends in efficacy for both rPFS and OS with no new safety signals observed.

In general, the baseline characteristics were generally well balanced between treatment arms and between the Asian subset and the global ITT population. Compared with the global ITT population, Asian patients in the olaparib arm had a lower median weight, higher rate of metastatic disease at initial diagnosis, higher median PSA at baseline and higher rate of visceral metastases. However, these differences in the baseline characteristics between the treatment arms in the Asian subset and between the Asian subset and global ITT population do not appear to have had any meaningful effect on the rPFS and OS results.

Median duration of treatment exposure in the Asian subset was approximately twice as long in the olaparib arm as in the control arm, which may have contributed to the higher incidence of certain AEs (e.g. anemia, nausea, decreased appetite and back pain) in the olaparib group. Overall, however, the safety and tolerability profile of olaparib in Asian patients, such as the incidence of AEs leading to treatment discontinuation or dose reduction, was generally similar to that reported previously in the global PROfound study population (2,7), suggesting that the slight imbalances in the baseline characteristics between treatment arms for the Asian subsets did not impact the safety of olaparib in these patients. These findings are consistent with recent Phase III data from the OlympiAD study examining the safety and tolerability of olaparib between Asian and Caucasian populations in metastatic breast cancer (13).

It has been proposed that there may be race-related differences in tolerability and response to anticancer drugs in patients with mCRPC. For example, Japanese physicians have long noted that the standard, approved doses of many agents are intolerable to Japanese patients, with a number of studies in various tumor types demonstrating a higher risk of grade ≥ 3 neutropenia in Asian patients than in their Western counterparts (14–17). Our data showed no marked contrasts in incidences of all-grade AEs and grade ≥ 3 AEs between Asian patients and the global PROfound study population (2,7). In the Asian subset of the PROfound study, the greatest contrast in incidence of grade ≥ 3 AEs in the olaparib arm between Asian patients and the global study population was decreased white blood cell count, although neutropenia was not more frequent in Asian patients (2). The finding of similar safety profiles in Asian and global ITT populations supports the safety of olaparib treatment in global and Asian populations. This is consistent with previous studies reporting similar pharmacokinetics of olaparib between Asian and Western patients (18).

In conclusion, the PROfound study is the largest PARPi trial to date to include an Asian population for prostate cancer, and the data presented here suggest a greater clinical benefit with olaparib than with abiraterone or enzalutamide in Asian patients with mCRPC with alterations in HRR genes who had disease progression on prior NHA. Olaparib was generally well tolerated in Asian patients, and this was characterized by low discontinuation rates. The safety profile of olaparib in the Asian subset was broadly consistent with that observed in the global PROfound study population.

## Funding

This work was supported by AstraZeneca and is part of an alliance between AstraZeneca and Merck Sharp & Dohme Corp., a subsidiary of Merck & Co., Inc., Kenilworth, NJ, USA.

## Disclosures

N.M.: Advisory role for AstraZeneca, Janssen, Bayer, Sanofi, Merck Sharp & Dohme, Roche, Amgen and Lilly; speaker bureau/expert testimony for AstraZeneca, Janssen and Sanofi; grants and institution funding from AstraZeneca, Janssen, Bayer, Sanofi, Merck Sharp & Dohme, Roche, Eisai, Takeda, Amgen, Pfizer and Lilly.

K.N.: Lecture fees/honoraria for Bayer, Novartis and Astellas Pharma; grants and institution funding from Bayer.

S.K.: Nothing to disclose.

J.Y.J.: Nothing to disclose.

H.U.: Advisory roles for AstraZeneca, Janssen Oncology, Bayer, Astellas, Sanofi, Takeda, Chugai and Amgen; and honoraria from Daiichi Sankyo, Kissei, MSD, Nihon Kayaku, Nihon Shinyaku, Kyowa Hakko Kirin Co., Ltd and Fujifilm-Toyama Chemical.

T.G.: Nothing to disclose.

T.G.K.: Advisory role for Bayer and Pfizer.

M.S.: Research grant (principal investigator, collaborator or consultant; pending and received grants) from Astellas; speakers’ bureau/honoraria for Janssen Pharmaceutical K.K., Takeda Pharmaceutical K.K., AstraZeneca, Nippon Shinyaku Co., Ltd, Pfizer Inc., Kissei and Bristol-Myers Squibb.

M.K.: Personal fees from AstraZeneca and Janssen; grants and institution funding from AstraZeneca.

S.S.W.: Nothing to disclose.

S.T.P.: Nothing to disclose.

C.H.C.: Nothing to disclose.

T.F.: Employment with AstraZeneca.

M.N.: Employment with AstraZeneca.

L.S.: Employment with Merck Sharp & Dohme Corp., a subsidiary of Merck & Co., Inc., Kenilworth, NJ, USA; and owns stock in Merck & Co., Inc., Kenilworth, NJ, USA.

M.D.: Employment with AstraZeneca.

M.H.: Consulting or advisory role for Bristol-Myers Squibb, Daiichi Sankyo Company, Janssen and Pfizer; honoraria for educational functions/lectures for Astellas Pharma, AstraZeneca, MLI PeerView, OncLive, PER, Phillips Gilmore Oncology, Projects in Knowledge, Research to Practice, Sanofi/Genzyme, UroToday, Precisca, Merck, Reach MD and Web MD; grants and institution funding from AstraZeneca, Bayer, Genentech, PCCTC, Pfizer (UM-Inst) and Arvinas.

J.d.B.: Grants and personal fees from AstraZeneca during the conduct of the study; outside the submitted work: personal fees and non-financial support from Astellas Pharma; grants, personal fees and non-financial support from AstraZeneca; personal fees from Genentech/Roche, Pfizer, Bayer, Boehringer Ingelheim, Merck Serono, Merck Sharp & Dohme and Janssen; personal fees and non-financial support from Sanofi; non-financial support from Genmab, GlaxoSmithKline, Orion Pharma GmbH, Qiagen, Taiho Pharmaceutical and Vertex; and personal and other fees from Cellcentric, Daiichi, GSK, Menarini/Silicon Biosystems and Sierra Oncology. In addition, Dr de Bono has a patent for abiraterone and steroids for the treatment of prostate cancer, with royalties paid to his institution, The Institute of Cancer Research. The Institute of Cancer Research also has a patent for PARP inhibitors and DNA repair defects, with royalties paid to The Institute of Cancer Research.

## Supplementary Material

PROfound_Asian_subgroup_ms_supplementary_appendix_hyac015
