# Supplementary material for: Olaparib in patients with mCRPC with homologous recombination repair gene alterations: PROfound Asian subset analysis
Source: Jpn J Clin Oncol. 2022 Feb 28;52(5):441–8. doi: 10.1093/jjco/hyac015 (PMC9071396; doi:10.1093/jjco/hyac015)
Supplement: PROfound_Asian_subgroup_ms_supplementary_appendix_hyac015 [file profound_asian_subgroup_ms_supplementary_appendix_hyac015.doc]

**Supplementary Appendix**

Supplement to: Matsubara N, Nishimura K, Kawakami S, *et al*. Olaparib in patients with mCRPC with homologous recombination repair gene alterations: PROfound Asian subset analysis. *Jpn J Clin Oncol* 2022;

## Table S1. Prevalence of HRR mutations (HRRm) in screened patients.

|  | **Japan  (n=302)** | **South Korea  (n=118)** | **Taiwan  (n=124)** |
| --- | --- | --- | --- |
|  | *Number of patients with HRRm (% successful results)* | | |
| ***BRCA1*** | 6 (2.0) | 1 (0.8) | 1 (0.8) |
| ***BRCA2*** | 25 (8.3) | 23 (19.5) | 15 (12.1) |
| ***CDK12*** | 37 (12.3) | 15 (12.7) | 11 (8.9) |
| ***ATM*** | 28 (9.3) | 7 (5.9) | 7 (5.6) |
| ***CHEK2*** | 0 | 0 | 1 (0.8) |
| ***PPP2R2A*** | 2 (0.7) | 0 | 4 (3.2) |
| ***PALB2*** | 1 (0.3) | 2 (1.7) | 0 |
| ***BRIP1*** | 0 | 2 (1.7) | 1 (0.8) |
| ***BARD1*** | 1 (0.3) | 0 | 1 (0.8) |
| ***RAD54L*** | 0 | 0 | 1 (0.8) |
| ***RAD51B*** | 1 (0.3) | 0 | 1 (0.8) |
| ***RAD51D*** | 1 (0.3) | 0 | 0 |
| ***CHEK1*** | 0 | 0 | 0 |
| ***FANCL*** | 0 | 0 | 0 |
| ***RAD51C*** | 0 | 0 | 0 |
| **Total HRRm** | 101 (33.4) | 48 (40.7) | 39 (31.5) |
